# High stillbirth rate in a swine farm in Vietnam and associated risk factors

**DOI:** 10.5455/javar.2022.i564

**Published:** 2022-01-14

**Authors:** Do Thi Kim Lanh, Nguyen Hoai Nam

**Affiliations:** Department of Animal Surgery and Theriogenology, Faculty of Veterinary Medicine, Vietnam National University of Agriculture, Hanoi, Vietnam

**Keywords:** Birth weight, farrowing duration, gestation length, litter size, parity, stillborn piglets

## Abstract

**Objective:**

The information about risk factors for a high stillbirth rate in piglets is inadequate. Therefore, this study was conducted to determine important risk factors for an extremely high stillbirth rate in a commercial pig farm in Vietnam.

**Materials and Methods:**

This study included 628 piglets and 45 Landrace × Yorkshire sows. Data including parity number, gestation length (GL), litter size (LS), piglet’s gender, stillbirth, birth order, birth interval (BI), cumulative farrowing duration (CFD), birth weight (BW), crown-rump length (CRL), body mass index, and ponderal index (PI) were collected. To deal with hierarchical data where several piglets might be born from a sow, Generalized Linear Mixed Models (GLMMs) were used to examine the association between stillbirth and investigated risk factors.

**Results:**

The stillbirth rate was 14.3%, and the incidence of stillbirth at the litter level was 68.9%. The final multivariate GLMM selected eight factors, including CFD, BI, CRL, BW, PI, GL, LS, and parity, as significant risk factors for stillbirth in the piglet. CFD >90 min, BI > 30 min, CRL <25 cm, BW <1.0 kg, PI <50, GL <114 days, LS >13, and parity 5–8 were associated with increased stillbirth. The final model explained 50.1% of the variation of stillbirth, in which fixed factors explained 43.6% of the variation.

**Conclusion:**

The present study indicated that the stillbirth rate in the investigated pig farm was very high, and several factors simultaneously contributed to the situation. Selection for optimal size and shape of piglets, careful supervision of parturition, and replacement of old sows should be some of the practical approaches to reduce the stillbirth rate.

## Introduction

Stillbirths are fully formed piglets born dead [1]. About 15% of stillborn piglets die before the onset of parturition, and 70% of them die during the farrowing process [2]. In recent decades, intensive genetic selection has resulted in the production of highly prolific sow lines capable of producing 40 piglets per sow per year [3]. Unfortunately, an increased litter size (LS) may reduce individual birth weight (BW) and increase farrowing duration, resulting in harmful effects on piglets’ probability of perinatal survival [4,5].

Stillbirth causes significant loss to pig-raising systems and raises animal welfare issues. An increased stillbirth rate has been reported in large LSs, long farrowing durations, high parity sows, and short gestation lengths (GL) [6]. Recently, several important piglet characteristics related to stillbirth, i.e., BW, body mass index (BMI), ponderal index (PI), birth interval (BI), birth order (BO), and cumulative farrowing duration (CFD), have been identified [5,7–10].

The stillbirth rate usually accounts for 5%–10% of the total number of born piglets [11] and varies between herds [12]. Some farms may incur a very high stillbirth rate (12%) [13]. Thus, it is reasonable to hypothesize that several factors may simultaneously contribute to the stillbirth status on such farms. However, the information about risk factors for a high stillbirth rate is still inadequate, and previous studies have never evaluated the effect of piglet factors on an extremely high stillbirth status. Therefore, the present study investigated the effects of various risk factors, including piglet, sow, and farrowing factors, on stillbirth in piglets on a farm with a high stillbirth rate.

## Materials and Methods

### Ethical approval

No animal samples were used in this study, and all animal handling practices followed the guidelines for the treatment of animals in behavioral research and teaching.

### Animals and housing

This study was conducted on a farm in Hung Yen province, Vietnam, from June to November 2019. This farm did not have a history of any outbreaks of abortion or stillbirth. Investigated Landrace × Yorkshire crossbred sows were in parity 1 to 8. After weaning, sows were fed 3.0–3.5 kg of industrialized feed. Sows were artificially inseminated twice with Duroc boars’ semen. During pregnancy, sows were fed 2.0 to 3.0 kg of industrialized feed. Sows *ad libitum* accessed to water provided through a bite nipple system. Sows were vaccinated against classical swine fever, porcine reproductive and respiratory syndrome, foot and mouth disease, pseudorabies, and parvovirus disease. Deworming was done twice per year. Sows were bathed once or twice a day, depending on ambient temperature. Pregnant sows were kept in individual gestation crates sized 220 × 60 cm. About a week before the anticipated farrowing date, sows were reallocated into individual farrowing crates sized 220 × 180 cm. A farrowing crate was divided into sow’s place (220 × 60 cm) and piglets’ place. An incubator heated with an infrared light was placed at one corner of the farrowing crate for newborn piglets. After being born, piglets were dried with clothes and breastfed within 1 hour of delivery. 

### Data collection

Data of 628 piglets and 45 Landrace × Yorkshire sows were recorded during the study period. Parity number and date of insemination were collected from sow cards, and GL was calculated as the interval between the insemination date and the farrowing date. Sows were monitored for signs of nest building, vulva swelling, and milk let down for preparation of farrowing supervision. Sows were fully supervised from at least the birth of the first piglets to the birth of the last piglets. The time of delivery of all individual piglets was recorded. The BI of each piglet was defined as the period between the births of two successive piglets. The CFD of each piglet was the interval between the birth of a given piglet and the birth of the first piglet. Therefore, the first piglets’ BI and CFD were not available. Piglets were weighed with a 5 gm precision digital scale and measured with a millimeter-scaled tape measure for crown-rump length (CRL). BW and CRL were measured before colostrum feeding and lasted less than 40 sec to avoid stressing the animals under study. The following equations were used to calculate BMI and PI from BW and CRL measurements: BMI = [BW (kg)/ CRL (m)2] and PI = [BW (kg)/ CRL (m)3]. Mummified piglets were born dead with a clear sign of body decomposition, autolysis, and brown/black color. Stillborn piglets were born dead with no sign of autolysis. The number of piglets born alive, stillborn, and mummified made up the LS. 

### Data analysis

Descriptive statistics were derived from all the available data of 628 piglets and 45 sows (Statistical Package for the Social Sciences, Version 22.0) (Table 1). For risk analysis, 45 first-born, 11 mummified piglets, and 4 piglets that did not have information on BW and CRL were discarded, leaving 568 piglets with complete details. Parity was divided into 1, 2–4, and 5–8; GL was divided into 112–113, 114–116, and 117–118 days; BO was divided into 2-5, 6-10, and 11-20; LS was divided into 5-13 and 14-20; BI was divided into 30 and >30 min; CFD was divided into 90, 90–240, and > 240 min; crown–rump length was divided into 25, 25–29, and >29 cm; BW was divided into <1.0, 1.0-1.8, and >1.8 kg; BMI was divided into <16 and >16; PI was divided into < 50 and > 50. Spearman’s correlation was used to quantify the associations between independent variables (Table 2). To account for the hierarchical character of the data where piglets were nested in litters, a Generalized Linear Mixed Model (GLMM) was used to determine significant risk factors for stillbirth in the piglet. In all models, the sow was fitted as a random factor to consider the potential difference in litters. In contrast, parity, GL, LS, BO, BI, CFD, BW, CRL, BMI, PI, and piglets’ gender were fitted as independent variables. The risk analysis was conducted in the following two steps. First, univariate GLMMs were undertaken to determine risk factors significant at *p *< 0.1 (Table 3). Second, combinations of different important factors were analyzed with different multivariate GLMMs to establish the final model that best explained the variation of stillbirth (Table 4).

**Table 1.** Descriptive statistics of 628 piglets born from 45 Landrace × Yorkshire sows on a farm in Vietnam.

Parameters	*n*	Mean ± SD/percentage
P	45	2.73 ± 1.8
GL (day)	45	114.9 ± 1.36
LS	45	14.6 ± 2.4
FD (min)	45	263.7 ± 176.8
CRL (cm)	617	28.3 ± 3.4
BW (100 gm)	617	14.5 ± 3.9
BMI	617	18.3 ± 5.3
PI	617	66.6 ± 42.8
BI (min)	582	20.7 ± 42.8
Stillbirth rate^a^ (%)	617	14.3 (88/617)
Mummy rate (%)	628	1.8 (11/628)
Incidence of stillbirth at litter level (%)	45	68.9 (31/45)

^a^ Exclusion of 11 mummified fetuses.

SD: Standard deviation; P: Parity, GL: Gestation length, LS: Litter size, FD: Farrowing duration, CRL: Crown-rump length; BW: Birth weight; BMI: Body mass index; PI: Ponderal index; BI: Birth interval.

**Table 2.** Correlation between potential risk factors for stillbirth in 568 piglets born from 45 Landrace × Yorkshire sows on a farm in Vietnam.

	*P*	GL	LS	BO	Gender	CRL	BW	BMI	PI	BI
GL	−0.07									
LS	0.01	0.096^a^								
BO	−0.03	0.05	0.220^b^							
GD	0.00	0.00	0.02	0.04						
CRL	0.08	0.03	−0.123^b^	−0.06	0.06					
BW	0.07	0.111^b^	0.06	0.089^a^	−0.01	−0.02				
BMI	0.191^b^	−0.08	−0.195^b^	−0.108^b^	0.108^b^	0.258^b^	−0.285^b^			
PI	0.07	−0.097^a^	−0.08	−0.116^b^	0.04	−0.02	−0.128^b^	0.582^b^		
BI	0.06	0.087^a^	−0.02	0.03	−0.03	−0.01	0.03	−0.04	−0.04	
CFD	0.07	0.08	0.121^b^	0.673^b^	0.04	−0.01	0.04	0.02	−0.04	0.257^b^

P: Parity, GL: Gestation length, LS: Litter size, BO: Birth order, CRL: Crown-rump length, BW: Birth weight, BMI: Body mass index, PI: Ponderal index, BI: Birth interval, CFD: Cumulative farrowing duration, GD: Gender.

a denotes significance level < 0.05.

b denotes significance level < 0.01.

## Results

Descriptive statistics are shown in Table 1. GL, LS, BI, and farrowing duration were 114.9 ± 1.36 days, 14.6 ± 2.4, 20.7 ± 42.8 min, and 263.7 ± 176.8 min, respectively. CRL, BW, BMI, and PI were 28.3 ± 3.4 cm, 1.45 ± 0.39 kg, 18.3 ± 5.3, and 66.6 ± 42.8, respectively. The stillbirth rate was 14.3%, and the incidence of stillbirth at the litter level was 68.9%. The mummified rate was 1.8%.

Table 2 presents Spearman’s correlations between potential risk factors. Apart from correlations between BO and CFD (Spearman’s rho = 0.673; *p *< 0.01), and between BMI and PI (Spearman’s rho = 0.582, *p *< 0.01), all other correlations were low (Spearman’s rho <0.3). Due to high correlations, some factors could not be selected in the same GLMM. Univariate analysis demonstrated that, except for piglets’ gender, all other factors significantly affected the stillbirth of piglets (Table 3).

The final GLMM that best explained the variation of stillbirth selected eight factors, including CFD, BI, CRL, BW, PI, GL, LS, and parity, as significant factors for stillbirth (Table 4). CFD, BI, and LS were positively associated with stillbirth. In contrast, the PI was negatively associated with stillbirth. CRL, BW, and parity had a curvilinear correlation with stillbirth. A GL of 112–113 days induced a higher risk of stillbirth than that of 114–116 days. The final multivariate model explained 50.1% of the variation of stillbirth, in which fixed factors explained 43.6%. The Hosmer–Lemeshow goodness of fit test showed a good fit between the observed and expected outcome (*p *= 0.640).

## Discussion

This is the first study to report simultaneous effects of various factors, including piglet ones, on an extremely high stillbirth rate in piglets. The stillbirth rate of piglets (14.3%) in this study was very high in comparison with results that have been reported by others (4.1%–7.5%) [10–17]. We recently reported a stillbirth rate that varied between 5.2% and 8.4% in different populations of piglets in Vietnam [4,5,10,18]. The incidence of stillbirth at the litter level in this study (68.9%) was also higher than any data ever reported (27.8%–60.2%) [5,10–11,13,16–18]. The result suggested that stillbirth was really an economic and animal welfare problem on the investigated farm.

**Table 3.** Univariate GLMM analysis of potential risk factors for stillbirth of 568 piglets born from 45 Landrace × Yorkshire sows on a farm in Vietnam.

Covariates	Stillbirth rate	CI; 95% CI; P
*P* = 2−4	9.0 (25/279)	1
*P* = 1	13.8 (23/167)	1.96; 0.61−6.29; 0.256
*P* = 5−8	27.0 (33/122)	4.38; 1.27−15.10; 0.019
GL = 114−116 days	9.4 (38/404)	1
GL = 117−118 days	12.3 (7/57)	1.41; 0.28−7.12; 0.677
GL = 112−113 days	33.6 (36/107)	5.98; 1.92−18.62; 0.002
LS = 5−13	6.3 (12/189)	1
LS = 14−20	18.2 (69/379)	3.76; 1.23−11.44; 0.020
BO = 2−5	9.6 (17/178)	1
BO = 6−10	12.4 (26/210)	1.41; 0.68− 2.95; 0.356
BO >10	21.1 (38/180)	2.94; 1.42−6.09; 0.004
M	14.8 (42/283)	1
F	13.7 (39/285)	094; 0.54−1.63; 0.831
BI < 30 min	11.5 (56/489)	1
BI >30 min	31.6 (25/79)	3.05; 1.50−6.19; 0.002
CFD < 90 min	7.8 (22/282)	1
CFD = 90−240 min	15.9 (37/232)	2.53; 1.32−4.85; 0.005
CFD > 240 min	40.7 (22/54)	4.89; 1.92−12.45; < 0.001
CRL = 25−29 cm	9.5 (23/242)	1
CRL > 29 cm	18.7 (45/241)	1.53; 0.72−3.23; 0.267
CRL < 25 cm	15.3 (13/85)	2.34; 0.94−5.81;0.066
BW = 1.0−1.8 kg	9.2 (32/349)	1
BW >1.8 kg	22.3 (25/112)	1.71; 0.77−3.79; 0.184
BW < 1.0 kg	22.4 (24/107)	4.29; 2.06−8.96; < 0.001
BMI > 16	18.2 (36/198)	1
BMI < 16	12.2 (45/370)	0.35; 0.18−0.68; 0.002
PI < 50	30.3 (33/109)	1
PI > 50	14.3 (81/568)	0.22; 0.11−0.44; < 0.001

P: Parity, GL: Gestation length, LS: Litter size, BO: Birth order, CRL: Crown-rump length, BW: Birth weight, BMI: Body mass index, PI: Ponderal index, BI: Birth interval, CFD: Cumulative farrowing duration, M: Male, FM: Female. CI: Confidence interval.

A positive association between CFD and BI and stillbirth has been well-established in previous studies [5,7,19–21]. Uterine contraction forces the expulsion of fetuses, however, it reduces the blood flow to the placenta, resulting in potential hypoxia and stress in piglets. Therefore, an increase in CFD and BI results in increased hypoxia and stress, causing an elevated risk of stillbirth in piglets. Previous studies showed increased stillbirth rate when BIs were prolonged by more than 60 min [20] or 90 min [19]. In this study, the effect of the BI was apparent earlier (>30 min), which may be one of the reasons for the high stillbirth rate in the investigated animals.

This study indicated that body size and shape are important factors for stillbirth in piglets. These results corroborate the findings of several authors [8,5,10]. Furthermore, this study also indicated that PI was more important than other conformation characteristics (CRL, BW, and BMI) in explaining stillbirth. Similar results were also previously reported [5,8,10]. Small piglets (<1.0 kg and/or <25 cm) had a lower blood concentration of hemoglobin [22] and were at an increased risk of asphyxia, resulting in an elevated risk of stillbirth. Piglets with low PI and BMI were usually small and disproportionate. These piglets might have suffered from uterine growth retardation [23] that might have predisposed them to substandard nutrition [24] and diminished cellular immunity [25], and subsequently were more likely to be stillborn.

**Table 4.** Multivariate GLMM analysis of potential risk factors for stillbirth of 568 piglets born from 45 Landrace × Yorkshire sows on a farm in Vietnam.

Covariates	CI; 95% CI; P
CFD < 90 min	1
CFD = 90−240 min	2.47; 1.25−4.89; 0.009
CFD > 240 min	3.84; 1.36−10.84; 0.011
BI < 30 min	1
BI > 30 min	3.08; 1.41−6.73; 0.005
CRL = 25−29 cm	1
CRL > 29 cm	2.19; 0.91−5.30; 0.081
CRL < 25 cm	3.48; 1.27−9.54; 0.015
BW = 1.0−1.8 kg	1
BW > 1.8 kg	2.05; 0.88−4.79; 0.096
BW < 1.0 kg	3.05; 1.25−7.45; 0.014
PI < 50	1
PI > 50	0.25; 0.02−0.53; <0.001
GL = 114−116 days	1
GL = 117−118 days	0.86; 0.20−3.57; 0.830
GL = 112−113 days	3.68; 1.49−9.08; 0.005
LS = 5−13	1
LS = 14−20	3.79; 1.46−9.82; 0.006
*P* = 2−4	1
*P* = 1	1.51; 0.57−3.95; 0.401
*P* = 5−8	6.37; 2.30−17.63; <0.001

P: Parity, GL: Gestation length, LS: Litter size, CRL: Crown-rump length, BW: Birth weight, PI: Ponderal index, BI: Birth interval, CFD: Cumulative farrowing duration. CI: Confidence interval.

Marginal *R*_2_ = 43.6%; Conditional *R*_2_ = 50.1%. Hosmer–Lemeshow test had a *p*-value of 0.640.

The relationship between GL and stillbirth in this study was in agreement with Nam and Sukon [5,10]. Piglets born from sows with a gestation <114 days were smaller than those born from sows with a gestation of 114–116 days (1.42 *vs.* 1.47 kg). These piglets are also less mature than piglets born with a gestation >113 days. Therefore, piglets born from a short gestation are more susceptible to stillbirth.

The positive association between LS and stillbirth in this study is consistent with findings by Nam and Sukon [5,10] and may be attributable to the link between LS and BW. Increased LS reduced individual BW (Spearman’s rho = −0.111, *p *= 0.008). Furthermore, LSs >13 were more likely to be born before day 114 of gestation than those of <14 (20.3% *vs.* 15.9%). Taken together, an increase in LS resulted in an increased stillbirth rate.

The effect of parity on stillbirth has been demonstrated in several studies [5,18,26]. High parity sows had lower uterine contraction tone and longer farrowing duration [27]. Indeed, in this study, the CFD of piglets born from sows at parity 5–8 was significantly longer than that of piglets born from sows at parity 2–4 (134.9 *vs.* 104.5 min).

Previous studies showed that BO was an important risk factor for stillbirth [5,7–10]. In this study, the final model did not contain BO due to the high correlation between this factor and CFD. Piglets born with increased BO experienced an increased number of series of uterine contractions and, therefore, might suffer more stress and hypoxia and were more vulnerable to stillbirth.

There existed a limitation in this study since infectious agents were not tested. However, this issue should not affect the present results because infectious pathogens are reported to cause prepartum stillbirths, accounting for less than 15% of all stillbirths [2,6]. Also, the health status of the sows was good during the study period. Furthermore, all sows were vaccinated against porcine reproductive and respiratory syndrome and parvovirus disease.

## Conclusion

The present study’s data showed that the stillbirth rate on this investigated farm was very high, and many factors simultaneously contributed to this situation. Sows should be carefully supervised and assisted when the farrowing duration and BI are prolonged. Furthermore, the importance of body size and shape suggests that selection for optimal BW and shape of piglets should be a long-term approach to reduce stillbirth in piglets. Also, sow selection and replacement should avoid using old sows. Finally, the detrimental effect of early farrowing on stillbirth implies that farrowing induction protocols, if any, should not be done too early to minimize stillbirth in piglets.

## List of Abbreviations

BI: Birth interval, BO: Birth order, BW: Birth weight, BMI: Body mass index, CFD: Cumulative farrowing duration, CI: Confidence interval, CRL: Crown-rump length, GD: Gender, GL: Gestation length, LS: Litter size, P: Parity, PI: Ponderal index, SD: Standard deviation.
